# Heart Failure Induces Significant Changes in Nuclear Pore Complex of Human Cardiomyocytes

**DOI:** 10.1371/journal.pone.0048957

**Published:** 2012-11-12

**Authors:** Estefanía Tarazón, Miguel Rivera, Esther Roselló-Lletí, Maria Micaela Molina-Navarro, Ignacio José Sánchez-Lázaro, Francisco España, José Anastasio Montero, Francisca Lago, José Ramón González-Juanatey, Manuel Portolés

**Affiliations:** 1 Cardiocirculatory Unit, Research Center, Hospital Universitario La Fe, Valencia, Spain; 2 Heart Failure and Transplantation Unit, Cardiology Department, Hospital Universitario La Fe, Valencia, Spain; 3 Biochemistry Unit, Research Center, Hospital Universitario La Fe, Valencia, Spain; 4 Servicio de Cirugía Cardiovascular, Hospital Universitario La Fe, Valencia, Spain; 5 Cellular and Molecular Cardiology Research Unit, Department of Cardiology and Institute of Biomedical Research, University Clinical Hospital, Santiago de Compostela, Spain; 6 Cell Biology and Pathology Unit, Research Center, Hospital Universitario La Fe, Valencia, Spain; Harvard Medical School, United States of America

## Abstract

**Aims:**

The objectives of this study were to analyse the effect of heart failure (HF) on several proteins of nuclear pore complex (NPC) and their relationship with the human ventricular function.

**Methods and Results:**

A total of 88 human heart samples from ischemic (ICM, n = 52) and dilated (DCM, n = 36) patients undergoing heart transplant and control donors (CNT, n = 9) were analyzed by Western blot. Subcellular distribution of nucleoporins was analysed by fluorescence and immunocytochemistry. When we compared protein levels according to etiology, ICM showed significant higher levels of NDC1 (65%, p<0.0001), Nup160 (88%, p<0.0001) and Nup153 (137%, p = 0.004) than those of the CNT levels. Furthermore, DCM group showed significant differences for NDC1 (41%, p<0.0001), Nup160 (65%, p<0.0001), Nup153 (155%, p = 0.006) and Nup93 (88%, p<0.0001) compared with CNT. However, Nup155 and translocated promoter region (TPR) did not show significant differences in their levels in any etiology. Regarding the distribution of these proteins in cell nucleus, only NDC1 showed differences in HF. In addition, in the pathological group we obtained good relationship between the ventricular function parameters (LVEDD and LVESD) and Nup160 (r = −0382, p = 0.004; r = −0.290, p = 0.033; respectively).

**Conclusions:**

This study shows alterations in specific proteins (NDC1, Nup160, Nup153 and Nup93) that compose NPC in ischaemic and dilated human heart. These changes, related to ventricular function, could be accompanied by alterations in the nucleocytoplasmic transport. Therefore, our findings may be the basis for a new approach to HF management.

## Introduction

Clinical manifestations of heart failure (HF) are the result of cellular, molecular and interstitial changes that drive homeostatic control [1]. Heart failure has been associated fundamentally with changes in mitochondria [2], glycolytic enzymes [3], cytoskeletal proteins [4] and Ca^2+^ handling [5].

The nucleus plays a critical role in the overall behavior of the cell. Changes in the expression of nuclear components or mutations in nuclear proteins contribute to many human diseases, such as laminopathies, premature aging, and cancer [6]–[8]. However, there are few studies examining the importance of the nucleus, nucleolus and the nucleocytoplasmic transport in HF [9]–[10]. Recently, we reported the effect of this syndrome on the nucleocytoplasmic trafficking machinery, such as increased importin, exportin, Ran regulators and Nup62 levels in ischaemic and dilated human hearts [9]. Furthermore, we demonstrated in these same HF patients changes in the morphology and organization of nuclear components with overexpression of nucleolin protein [10].

We hypothesized whether we could also find any alteration in the nuclear pore complex (NPC) structure, the gateway connecting the nucleoplasm and cytoplasm. For this purpose, we selected six nucleoporins (Nups), representing structural features of NPC: transmembrane ring (NDC1), inner ring (Nup155), outer ring (Nup160), linker (Nup93), FG (Nup153) and peripheral (TPR) [11]. Most of these proteins have been associated with a number of diseases, such as cancer, disorders of the nervous and immune systems and cardiovascular diseases [12], but have never been analysed in human HF. Therefore, the main objective of this work was to study these different nucleoporins in left ventricle tissue from patients with ischaemic (ICM) and dilated cardiomyopathy (DCM).

## Methods

### Ethics Statement

All patients gave written informed consent to participate in the study. The project was approved by the local Ethics Committee (Biomedical Investigation Ethics Committee of “La Fe” Universitary Hospital of Valencia, Spain) and conducted in accordance with the guidelines of the Declaration of Helsinki [13].

**Table 1:** Patients characteristics according to HF aetiology.

	ICM (n = 52)	DCM (n = 36)
**Age (years)**	56±7	49±13**
**Gender male (%)**	92	78
**NYHA class**	3.4±0.4	3.3±0.5
**BMI (kg/m^2^)**	27±4	25±6
**Haemoglobin (mg/mL)**	13±2	13±2
**Haematocrit (%)**	40±6	40±7
**Total cholesterol (mg/dL)**	182±49	143±42**
**Prior hypertension (%)**	50	24*
**Prior smoking (%)**	85	64
**EF (%)**	23±7	21±8
**FS (%)**	13±4	11±4*
**LVESD (mm)**	56±9	66±11***
**LVEDD (mm)**	63±8	74±12***
**Left ventricle mass index (g/cm^2^)**	142±36	204±64***
**Duration of disease (months)**	62±57	70±57

Duration of disease from diagnosis of HF until heart transplant. BMI, body mass index; DCM, dilated cardiomyopathy; EF, ejection fraction: FS, fractional shortening; HYHA, New York Heart Association; ICM, ischaemic cardiomyopathy; LVEDD, left ventricular end-diastolic diameter; LVESD, left ventricular end-systolic diameter.

* p<0.05; **p<0.01; ***p<0.001.

### Source of Tissue

Experimental material was taken from a total of 88 explanted human failure hearts, 52 from patients with ICM and 36 from patients with DCM, undergoing cardiac transplantation. Clinical history, ECG, echocardiography, hemodynamic studies, and coronary angiography data were available on all patients. The clinical characteristics of the patients are shown in Table 1. All patients were functionally classified according to the New York Heart Association (NYHA) criteria and were receiving medical treatment following the guidelines of the European Society of Cardiology [14]. Nine non-diseased donor hearts were used as control (CNT) samples. The hearts were initially considered for cardiac transplantation but were subsequently deemed unsuitable for transplantation either because of blood type or size incompatibility. The cause of death was cerebrovascular accident or motor vehicle accident. All donors had normal left ventricular function and no history of myocardial disease or active infection at the time of transplantation.

**Figure 1:**
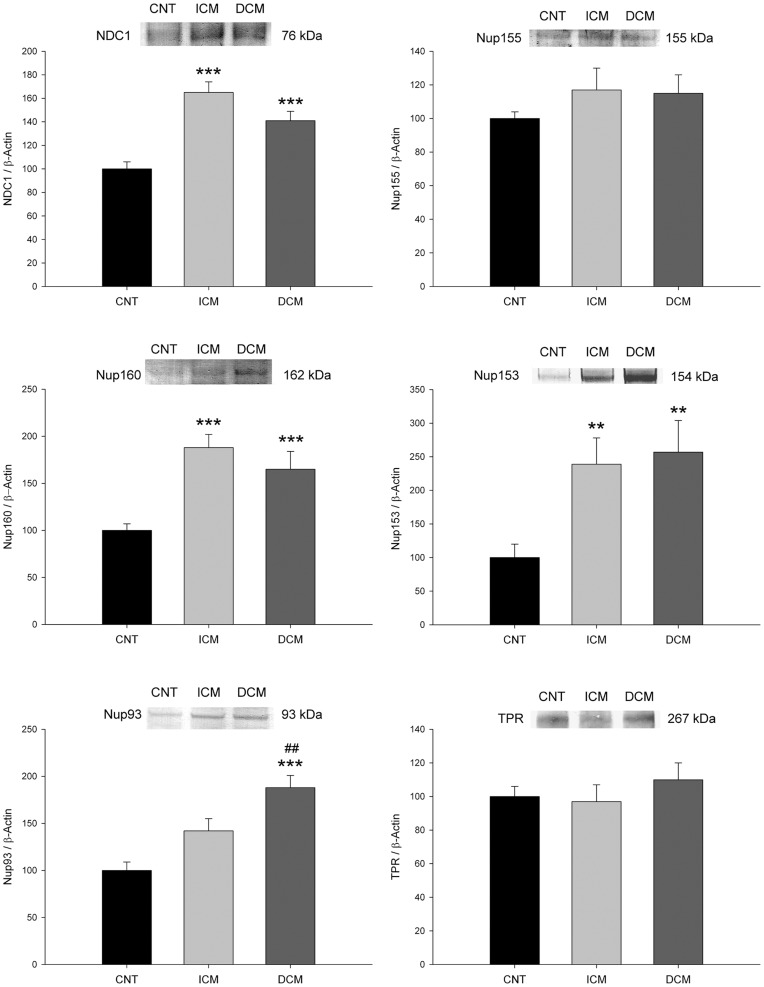
Influence of heart failure aetiology on the levels of nucleoporins. NDC1, Nup155, Nup160, Nup153, Nup93 and TPR were analysed by Western blot techniques. The values from the CNT group were set to 100. The data are expressed as mean ± SEM in arbitrary units (optical density) of six independent experiments. ICM, ischaemic cardiomyopathy; DCM, dilated cardiomyopathy; CNT, control. **p<0.01, ***p<0.0001 vs. CNT group. ^##^p<0.01, ICM vs. DCM group.

**Table 2:** Results of the expression of nuclear proteins in HF patients and controls.

	Controls (n = 9)	Patients (n = 88)	p value
**NDC1**	100±19	156±57	<0.0001
**Nup155**	100±10	112±37	NS
**Nup160**	100±20	177±93	<0.0001
**Nup153**	100±55	246±180	<0.0001
**Nup93**	100±25	160±85	= 0.023
**TPR**	100±18	104±51	NS

NDC1, Nuclear division cycle protein 1; Nup, nucleoporinas; TPR, translocated promoter region. The patients group includes ischaemic (ICM) and dilated cardiomyopathy (DCM).

Transmural samples were taken from near the apex of the left ventricle. The ICM, DCM, and CNT samples were flushed with 0.9% NaCl and stored at 4°C for a mean time of 4.4±3 h from loss of coronary circulation. All tissues were obtained with informed consent of patients.

**Figure 2:**
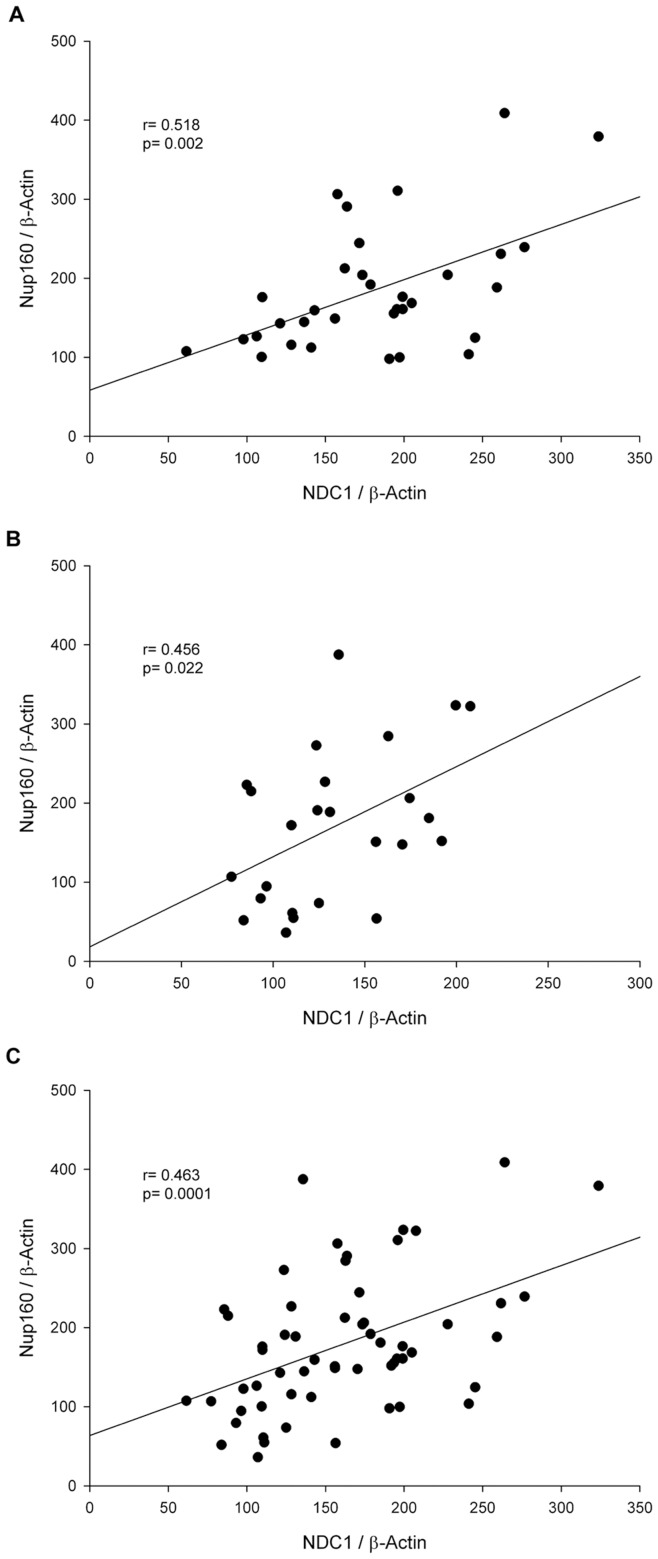
Relationships between Nup160 and NDC1 levels. Subjects with ICM (A), DCM (B) and all patients (C). Values are normalized to β-actin and finally to CNT group.

### Homogenization of Samples and Protein Determination

Twenty-five milligrams of frozen left ventricle was homogenized in the FastPrep-24 homogenizer (MP Biomedicals, USA) in specifically designed Lysing Matrix tubes, in a total protein extraction buffer (2% SDS, 10 mM EDTA, 6 mM Tris–HCl, pH 7.4) with protease inhibitors (25 µg/mL aprotinin and 10 µg/mL leupeptin). The isolation of nuclear and cytoplasmic protein fraction was obtained by NE-PER method (Thermo Scientific, USA). The homogenates were centrifuged and the supernatant was aliquoted. The protein content of the aliquot was determined by the Lowry method (Peterson’s Modification [15]) using bovine serum albumin (BSA) as standard.

**Figure 3:**
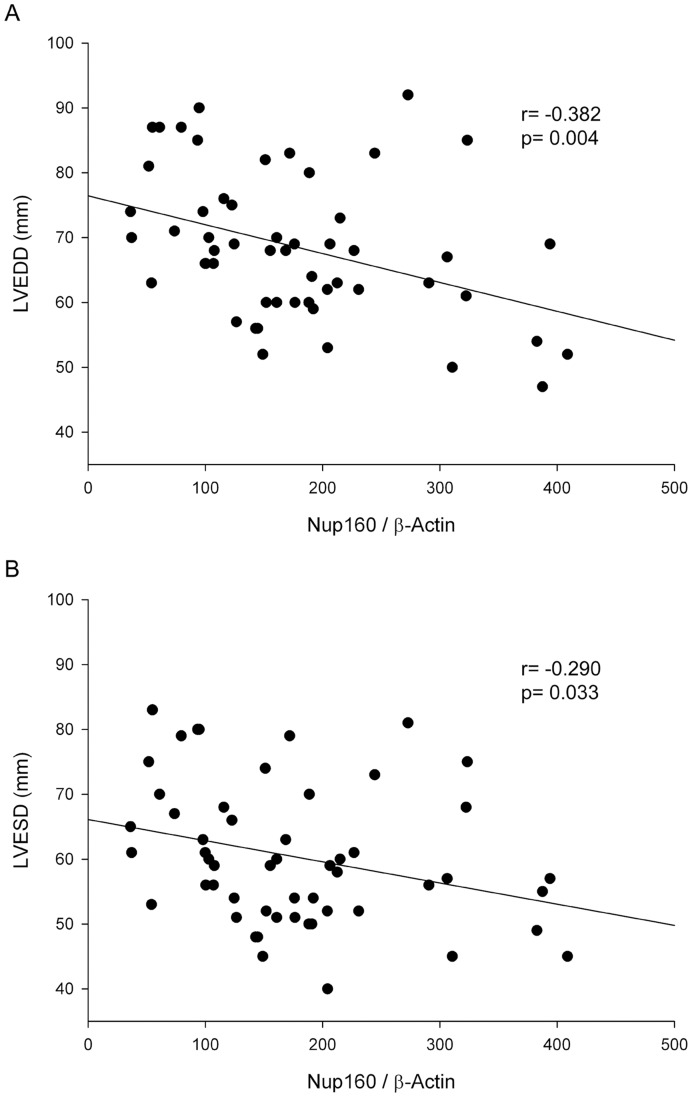
Relationships between Nup160 levels and ventricular function parameters. A) LVEDD (left ventricular end-diastolic diameter), B) LVESD (left ventricular end-systolic diameter) in HF patients group (ICM and DCM). Values are normalized to β-actin and finally to CNT group.

### Polyacrylamide Gel Electrophoresis and Western Blot Analysis

Samples were separated by Bis-Tris Midi gel electrophoresis with 4–12% polyacrylamide in a separate gel for NDC1, Nup155, Nup160, Nup153 and Nup93; and by Native Bis-Tris Mini gel electrophoresis with 4–16% polyacrylamide for TPR. After electrophoresis, the proteins were transferred from the gel to a PVDF membrane by the iBlot Dry Blotting System Ltd (Invitrogen, UK) for Western blot. The membrane was blocked all night at 4°C with 1% BSA in Tris-buffer solution containing 0.05% Tween 20 and then for 2 h with a primary antibody in the same buffer. For TPR the Western blot was performed in a BenchPro 4100 Card Processing Station (Invitrogen, Carlsbad, CA). The primary detection antibodies used were: anti-NDC1 rabbit polyclonal antibody (1/500 dilution), anti-Nup155 rabbit polyclonal antibody (1/800 dilution), anti-Nup160 rabbit polyclonal antibody (1/800 dilution), anti-Nup153 mouse monoclonal antibody (1/30 dilution), anti-Nup93 mouse monoclonal antibody (1/500 dilution) and anti-TPR mouse monoclonal antibody (1/1000 dilution), from Abcam (Cambridge, UK). Monoclonal anti-beta-actin antibody (1/1000 dilution) (Sigma-Aldrich, Missouri, USA) was used as loading CNT for each of the blots.

**Figure 4:**
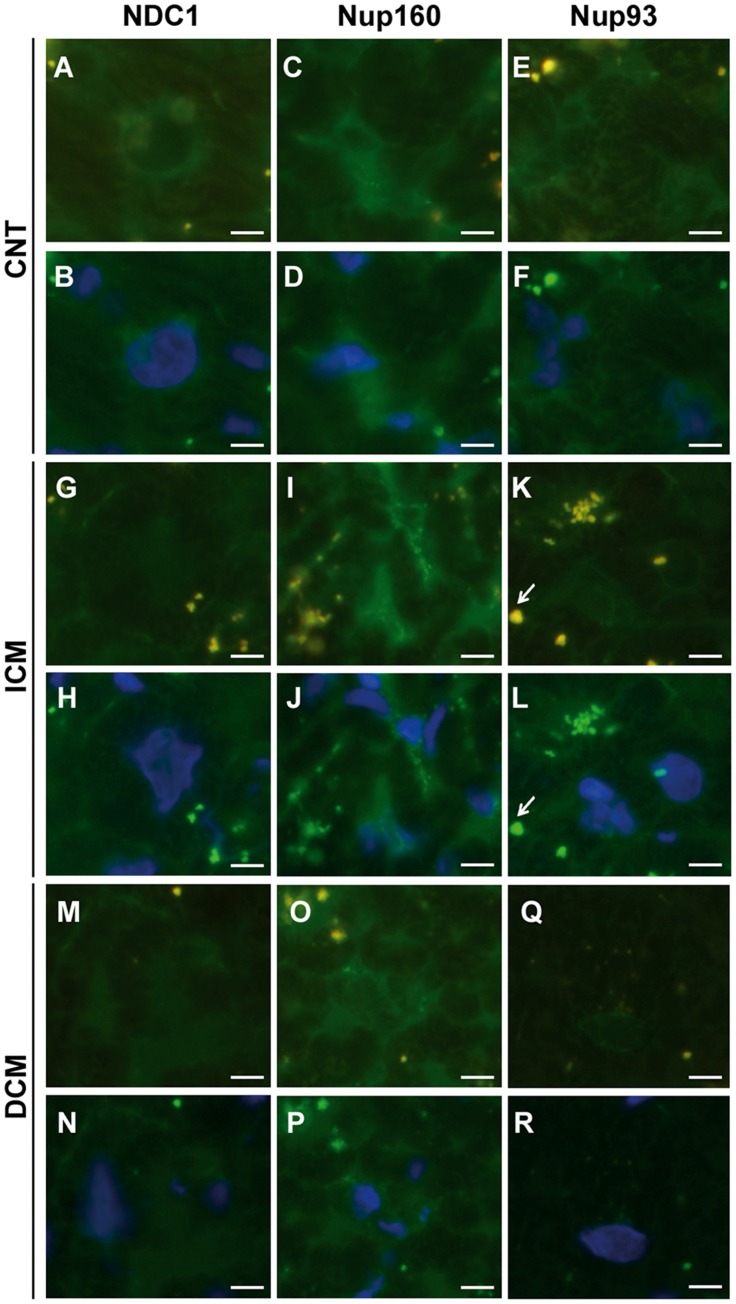
Effect of heart failure on cell distribution of some nucleoporins in left ventricular human cardiomyocytes. Inmunofluorescence staining with and without DAPI of NDC1, Nup160 and Nup93 according to heart failure aetiology, control (CNT) (Figure A,C and E, fluorescence; Figure B, D and F, DAPI A–F), ischaemic (ICM) (Figure G, I and K, fluorescence; Figure H, J and L, DAPI G–L) and dilated (DCM) (Figure M, O and Q, fluorescence; Figure N, P and R, DAPI M–R) groups. Arrows shows the immunofluorescence due to lipofuscin particles. Scale bar = 10 µm.

**Figure 5:**
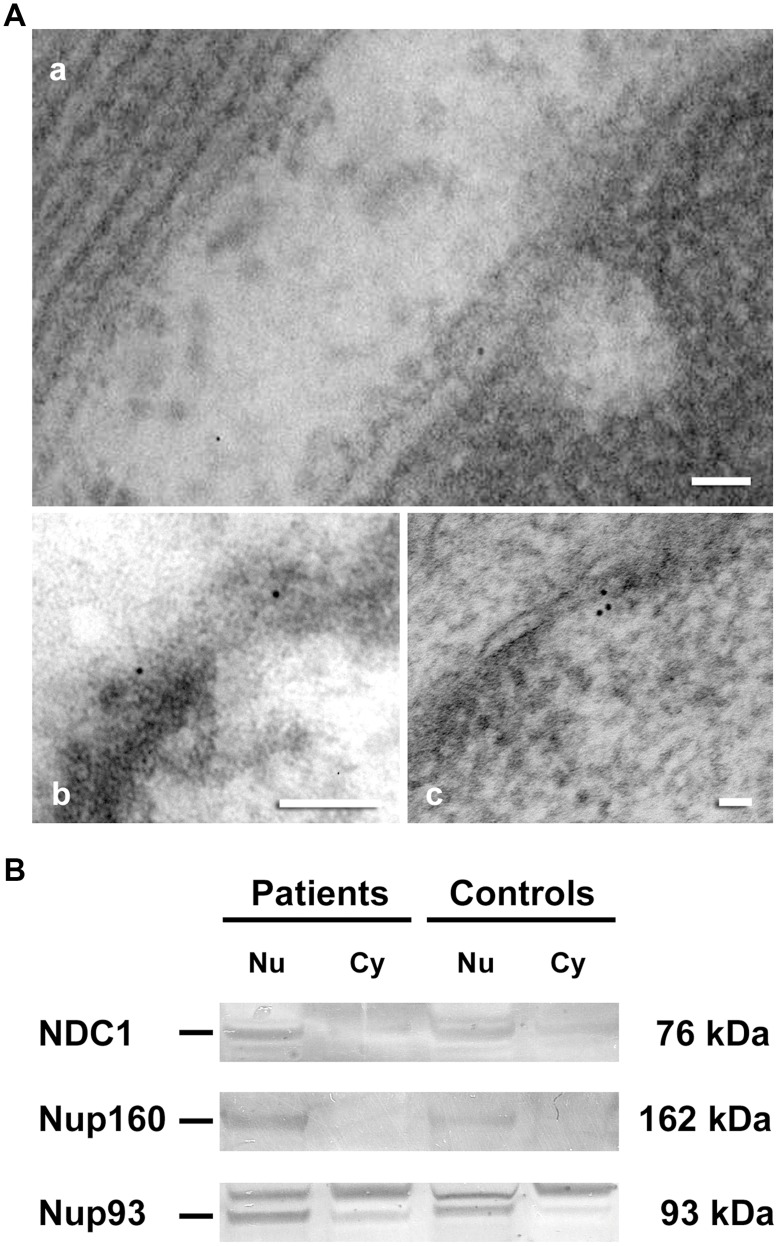
Immunolocalization of NDC1 in human cardiomyocytes and western blot of nucleoporins in nuclear and cytosolic fraction. (A) Electron micrograph, in all case, gold particles (10 nm) are over nuclear pore complex; in control (a), dilated (b) and ischaemic tissue (c). The labeling is increased in ischaemics. No labely of NDC1 was observed in other nuclear structure. Scale bar = 50 nm. (B) Western blot analysis of NDC1, Nup160 and Nup93 in nuclear (Nu) and cytosolic (Cy) fraction in controls and HF patients (ICM and DCM).

Then, the bands were visualized using an acid phosphatase conjugated secondary antibody and nitro blue tetrazolium/5-bromo-4-chloro-3-indolyl phosphate (NBT/BCIP) (Sigma-Aldrich, St. Louis, USA) substrate system. Finally, bands were digitalized using an image analyser (DNR Bio-Imaging Systems, Israel) and quantified by the Gel Capture (v.4.30) and the TotalLab TL-100 (v.2008) programs.

### Fluorescence Microscopy

To analyse protein distribution, frozen heart sections were transferred to glass slides and fixed in pure methanol for 15 min at −20°C. Then, samples were blocked with PBS containing 1% BSA for 15 min at room temperature (RT). After blocking, sections were incubated for 2 h at RT with the primary antibodies for Nup160 (1/250 dilution), Nup93 (1/250 dilution) and NDC1 (1/250 dilution) (described in Western blot analysis) in the same buffer solution, and then with Alexa-conjugated secondary antibody (Invitrogen, USA) for 60 min at RT [16]. Finally, sections were rinsed in PBS, mounted in Vectashield with DAPI (Vector, Burlingame, CA), and observed with an Olympus B-50 fluorescence microscope. The images were processed using ImageJ (v. 1.4.3.67) program (National Institute of Mental Health, Bethesda, Maryland, USA).

### Immunocytochemistry and Electron Microscopy

Myocardial samples (size 1 mm^3^) from left ventricle were fixed in a solution of 1.5% glutaraldehyde and 1% formaldehyde in 0.05 M cacodylate buffer, pH 7.4, for 1 h at 4°C. Then, samples were post-fixed in 1% OsO_4_ for 1 h at 4°C, dehydrated in ethanol and embedded in Epon 812. Ultra-thin sections measuring 80 nm were obtained and mounted on nickel grids and counter-stained with 2% uranyl acetate for 20 min and 2.7% lead citrate for 3 min [17]–[18].

For immunogold labeling ultra-thin sections were floated for 30 min on 0.1% BSA-Tris buffer (20 mM Tris- HCl, 0.9% NaCl, pH 7.4, containing 0.1% BSA, type V) and 2 h in a moist chamber at RT on sodium metaperiodate [19]. After rinses with bi-distilled water, the sections were incubated for 5 min with 3% hydrogen peroxide. The grids were rinsed again with bi-distilled water and incubated in a moist chamber overnight at RT with a primary antibody in the 0.1% BSA-Tris buffer. Anti-NDC1 rabbit polyclonal antibody (1/100 dilution), anti-Nup160 rabbit polyclonal antibody (1/50 dilution) and anti-Nup93 mouse monoclonal antibody (1/100 dilution) were used as primary detection antibodies separately.

After rinses with 0.1% BSA-Tris buffer, the sections were incubated in a moist chamber for 1 h at 37°C with 0.1% BSA-Tris buffer (containing 0.05% Tween-20) and a goat anti-rabbit IgG-gold antibody (10 nm, Sigma, 1/10 dilution) for NDC1 and Nup160, and a goat anti-mouse IgG-gold antibody (5 nm, Sigma, 1/10 dilution) for Nup93. After rinses with 0.1% BSA-Tris buffer and bi-distilled water, the sections were air dried and counterstained, first with uranyl acetate for 30 min and then with lead citrate for 30 sec. Finally, the grids were air dried completely.

For electron microscopy observation a Philips CM-100 was used, with magnifications ranging X4500–15000. A quantitative stereological analysis of the photomicrographs was performed to quantify the numerical density and distribution of proteins by iTEM FEI program (v. 5.0, 2008, Olympus Soft Imaging Solutions GmbH).

### Statistical Methods

Data are presented as the mean value ± SD for continuous variables and as percentages for discrete variables. The *Kolmogorov–Smirnov test* was used to analyse the distribution of the variables. Comparisons of clinical characteristics were achieved using *Student's t-test* for continuous variables and *Fisher exact test* for discrete variables. Comparisons of nuclear protein levels between different groups were performed using *Student’s t-test* for variables with a normal distribution and the *Mann–Whitney U test* for variables with a non-normal distribution. Nup93 concentrations exhibited a non-normal distribution and were log transformed (and proved to be normalized) before parametric correlation analysis. Finally, *Pearson’s correlation coefficient* was performed to analyse the association between variables. Significance was assumed as p<0.05. All statistical analyses were performed using SPSS software v. 11.5 for Windows (SPSS Inc.).

## Results

### Clinical Characteristics of Patients

We analysed 88 explanted human hearts from patients undergoing cardiac transplantation diagnosed with HF and 9 non-diseased donor hearts were used as CNT samples. Most of the patients were men (85%) with a mean age of 53±10 years, a mean NYHA functional classification of III–IV, and previously diagnosed with significant comorbidities including hypertension and hypercholesterolemia. Table 1 shows the clinical characteristics of patients according to aetiology of HF. The ICM group showed with respect to the DCM group a significant increase in age (p<0.01), prevalence of hypertension (p<0.05), cholesterol levels (p<0.01) and fractional shortening (FS) (p<0.05). Significant differences between the ICM and DCM patients, were also found in left ventricular end-systolic diameter (LVESD) (p<0.001), left ventricular end-diastolic diameter (LVEDD) (p<0.001) and left ventricle mass index (p<0.001); found higher values in the DCM group.

### Effects of HF on Levels of Nucleoporins

We analysed whether heart failure (HF) induced changes in the proteins of the NPC in cardiac tissue. For this, we determined NDC1, Nup155, Nup160, Nup153, Nup93 and TPR levels by Western blot analysis. When we compared protein levels between patients with HF and controls, NDC1, Nup160, Nup153 and Nup93 were significantly increased in pathological samples (156±57 *vs*. 100±19 arbitrary units (AU), p<0.0001; 177±93 *vs*. 100±20 AU, p<0.0001; 246±180 *vs*. 100±55 AU, p<0.0001; and 160±85 *vs*. 100±25 AU, p = 0.023, respectively). There were not any significant differences in Nup155 and TPR between HF patients and control group (Table 2). Then, we compared protein levels according to aetiology of HF; ICM hearts showed higher levels of NDC1 (65%, p<0.0001), Nup160 (88%, p<0.0001) and Nup153 (137%, p = 0.004) compared with control levels. In addition, DCM hearts showed significant differences in NDC1 (41%, p<0.0001), Nup160 (65%, p<0.0001), Nup153 (155%, p = 0.006) and Nup93 (88%, p<0.0001) compared with the control group. Nup155 and TPR did not show significant differences in their levels in any aetiology. We only observed differences in nuclear levels of Nup93 (142±88 vs. 188±71 AU, p = 0.001) comparing the two aetiologies studied (Figure 1).

Furthermore, we observed relationships between Nup155 and NDC1 proteins (r = −0.588, p = 0.044) in the ICM group (data not shown), and Nup160 and NDC1 in all groups: patients (r = 0.463, p = 0.0001), ICM (r = 0.518, p = 0.002) and DCM (r = 0.456, p = 0.022) (Figure 2).

Finally, we determined whether there was any relationship between NPC protein levels and the clinical characteristics shown in Table 1. In the pathological group (ICM and DCM) we obtained good relationships between the ventricular function parameters (LVEDD and LVESD) and Nup160 (r = −0.382, p = 0.004; r = −0.290, p = 0.033; respectively) (Figure 3). We also observed relationship between LVEDD and Nup160 in the DCM group (r = −0.425, p = 0.034; data not shown).

### Effects of HF on Distribution of Nuclear Proteins

The results of immunofluorescence studies for NDC1, Nup160 and Nup93 proteins were consistent with the increased level observed by Western blot. Regarding the distribution of the three proteins, NDC1 showed differences between the groups (Figure 4). In the control group the protein was distributed around the core surface while in the ischaemic and dilated groups there was diffuse distribution within the core. However, Nup160 and Nup93 presented the same distribution for all cases, patients and controls, around the nuclear area (Figure 4).

Immunocytochemistry studies confirmed the previous results of immunofluorescence. The Figure 5A shows an increase in immunogold labeling in NDC1 (10 nm particles) in dilated and ischaemic groups. A similar increase was observed in Nup160 for ischaemic and dilated groups and in Nup93 for dilated group (data not shown). In addition, we showed by Western blot the localization of these proteins in the nuclear fraction isolated from cardiac tissue of HF patients and controls (Figure 5B).

## Discussion

In the nucleus essential processes for cell life occur, such as gene expression, signal transduction or cell cycle progression [20]. The NPC selectively controls the passage of macromolecules such as RNA, ribosomes and proteins; therefore, it is an important way to control gene expression, signal transduction and cellular homeostasis [21]. Some human diseases such as cancer and immune or nervous system disorders are the result of changes in expression or mutations of the components of NPC [12]. However, previous works on this complex in cardiovascular diseases remain scant. Specifically, it has been observed that the nucleoporin Nup62 is increased in patients with ischaemic and dilated cardiomyopathy [9]. In addition, it has been found that a mutation in Nup155 leads to atrial fibrillation and sudden death [22]. We hypothesized that heart failure may change NPC structure and function. Therefore, in this work, to study the NPC in HF patients, we performed a mapping of this complex through the study of different representative proteins that at different levels of this structure make up: transmembrane ring (NDC1), inner ring (Nup155), outer ring (Nup160), FG nucleoporins (Nup153), linker nucleoporins (Nup93) and the periphery nucleoporins of the nuclear face (TPR).

The nucleoporins, besides performing a structural role in the NPC, are actively involved in nucleocytoplasmic transport [23]–[24]. In previous works, we demonstrated that HF influences the nucleocytoplasmic trafficking machinery of human hearts, affecting the morphology and organization of nuclear and nucleolar components. We observed significantly increased levels of importins, exportins, Ran regulators and Nup62 in patients with this pathology, and a different configuration and morphology of the NPC [9]. We also observed changes in expression of nucleolin in patients with ICM and DCM, and these changes correlate with ventricular function [10]. In another study of our group, we showed that HF causes different changes in nuclear structure and function, observing changes in the levels of lamin A and C, proteins that maintain the structure of the nuclear lamina and organization of proteins such as emerin [25]. In this study, we observed significant increases in levels of NDC1, Nup160, Nup153 and Nup93 in HF patients when compared to the control group. These results, together with the data observed in other studies, suggest a relationship between changes in nucleoporin levels and the alterations of the nucleocytoplasmic transport previously reported. It has been shown that overexpression of both Nup160 and Nup153 causes inhibition of mRNA export, observing nuclear accumulation of RNA poly(A)^+^; however, the import and export of proteins is not affected, so it seems that no nucleocytoplasmic transport reduction occurs, nor does occlusion or disassembly of the NPC [23]–[24]. The results obtained by Vasu *et al*. [24], showed that Nup160 could interact directly with specific factors or receptors involved in mRNA export and/or indirectly immobilize Nup153 in the pore, which has a region representing the docking site for mRNA molecules [23]. Thus, our results are consistent with these previous data.

Moreover, despite the significant increases in protein levels of Nup160 and Nup93 in HF patients compared with the control group, the immunofluorescence distribution patterns were similar in all the groups studied, these proteins being located in the nuclear envelope. Nup160 has an important structural role, participating in the initial stages of NPC assembly [26], and it is also involved in the transport of mRNA [24]. All these processes take place in the nuclear envelope, which could determine the subcellular localization of Nup160, especially in patients with HF where the nucleocytoplasmic transport is altered. Nup93 also has an important structural role since its depletion results in deformed pores, and together with the depletion of NDC1 causes a global disruption NPC [27]. Also, it has been seen that Nup93 is associated with important regions of the chromatin involved in transcriptional regulation [28]. As with Nup160, these are important functions that take place in the nuclear envelope and may determine the subcellular localization of Nup93. Stability in the subcellular localization of Nup93 has been confirmed in independent studies [28]–[29].

In addition, we have observed two distribution patterns in NDC1 protein. NDC1 is observed on the nuclear surface and inside the nucleus. Previous studies show a similar pattern in the control group of NDC1 in the nuclear surface [27]. NDC1 is an evolutionarily conserved transmembrane protein in most eukaryotes [27], necessary for the proper assembly of the NPC and its absence causes severe defects in the assembly and NPC biogenesis [30]–[31]. As described in *HeLa* cell cultures, we observed that NDC1 is located at the nuclear envelope in the CNT group [27]. However, we also observed a distribution within the nucleus in the ICM and DCM groups. It has been observed that in yeast increased Nup53p (hNup35) nucleoporin locates in the core, causing the accumulation of NDC1 in lamellar membranes inside the nucleus. In this sense, we believe that this protein could be related to Nup160, also a core protein increased in the HF patients group, sequestering NDC1 into the nucleus. Furthermore, the assembly of the components of the NPC is a complex and little known process that occurs in a space-time order [27]. Rasala *et al*. [26], propose a model for the early stages of NPC assembly in *Xenopus laevis*. According to this model, NDC1 is assembled into the NPC once the Nup160 complex is bound to the inner ring, which is consistent with the relationships that we have observed in this work between NDC1 and Nup160.

However the mechanism through which the expression of NPC proteins in HF patients increases is unclear. The human heart possesses a significant growth reserve, forming a large number of myocytes every year [32]. Probably cardiomyocytes turnover is promoted after HF, so that the newly formed myocytes posses larger quantity of NPC protein than older myocytes. This could be a possible mechanism for the increased expression of NPC proteins in HF. And our results are consistent with the compensatory response of cardiac stem cells, which differentiate and regenerate myocytes to counteract the dying cells.

Echocardiographic functional parameters are closely related to ventricular remodeling, a clear indicator of the HF progression. A long-term remodeling process becomes detrimental leading to a progressive cardiac decompensation [1]. We found that Nup160 was inversely related with ventricular function, in other words, higher levels of Nup160 are linked with left ventricular function improvement. These findings suggest that the levels of Nup160 could increase as a mechanism to prevent the heart from ventricular dysfunction. This observation could be interpreted as the “pseudo-normalization” due to decompensated turnover of cardiomyocytes in these patients [32].

One limitation of this study is the intrinsic variability of the samples, given they originate from human hearts, whose conditions (treatment they undergo) are not as standardized as those of studies using cell cultures. Furthermore, despite the results obtained, further studies are needed to determine the effect of HF on the NPC. Also, it would be interesting to study directly the nucleocytoplasmic transport in HF.

In summary, this study shows that patients with ischaemic and dilated cardiomyopathy present specific changes in the levels and distribution of the components of NPC. Our results show increased levels of various nucleoporins in patients undergoing heart transplantation when compared with controls. Besides, it showed a good relationship between NDC1 and Nup160 independently of the aetiology of HF, and an inverse association between left ventricular function parameters and Nup160. These changes could be accompanied by alterations in the nucleocytoplasmic transport. Therefore, our findings may be the basis for a new approach to HF management.
